# The Piranha Genome Provides Molecular Insight Associated to Its Unique Feeding Behavior

**DOI:** 10.1093/gbe/evz139

**Published:** 2019-07-08

**Authors:** Manfred Schartl, Susanne Kneitz, Helene Volkoff, Mateus Adolfi, Cornelia Schmidt, Petra Fischer, Patrick Minx, Chad Tomlinson, Axel Meyer, Wesley C Warren

**Affiliations:** 1 Physiologische Chemie, Biozentrum, University of Würzburg, Germany; 2 Comprehensive Cancer Center Mainfranken, University Clinic Würzburg, Germany; 3 Hagler Institute for Advanced Study, Texas A&M University; 4 Department of Biology, Texas A&M University; 5 Department of Biology, Memorial University of Newfoundland, St John’s, Canada; 6 Department of Biochemistry, Memorial University of Newfoundland, St John’s, Canada; 7 McDonnell Genome Institute, Washington University School of Medicine; 8 Chair in Zoology and Evolutionary Biology, University of Konstanz, Germany; 9 Bond Life Sciences Center, University of Missouri

**Keywords:** whole-genome sequencing, genome annotation, comparative genomics, RNA-seq transcriptome, energy homeostasis, starvation

## Abstract

The piranha enjoys notoriety due to its infamous predatory behavior but much is still not understood about its evolutionary origins and the underlying molecular mechanisms for its unusual feeding biology. We sequenced and assembled the red-bellied piranha (*Pygocentrus nattereri*) genome to aid future phenotypic and genetic investigations. The assembled draft genome is similar to other related fishes in repeat composition and gene count. Our evaluation of genes under positive selection suggests candidates for adaptations of piranhas’ feeding behavior in neural functions, behavior, and regulation of energy metabolism. In the fasted brain, we find genes differentially expressed that are involved in lipid metabolism and appetite regulation as well as genes that may control the aggression/boldness behavior of hungry piranhas. Our first analysis of the piranha genome offers new insight and resources for the study of piranha biology and for feeding motivation and starvation in other organisms.

## Introduction

Piranhas are well-known South-American fishes and paradigmatic representatives of Characids. Their widespread reputation comes from their predatory habits and remarkable feeding adaptations, which include large, sharp teeth, and the strongest bite force determined in any fish to date (Grubich et al. 2012).

**Fig. 1.**
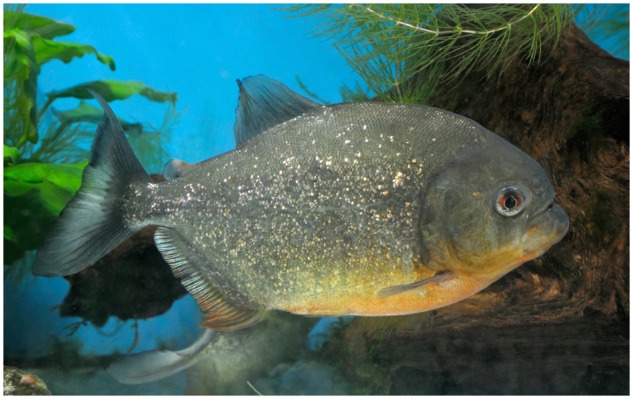
—The sequenced red-bellied Piranha, *Pygocentrus nattereri*, female.

Red-bellied piranha (*Pygocentrus nattereri*) (fig. 1) inhabits neotropical freshwater rivers of northeastern Brazil, and the Paraguay and Parana basins. Previous research on this species has primarily focused on attributes related to diet/feeding habits (Pauly 1994) and social and feeding behavior (Foxx 1972; Sazima and Machado 1990). Although it is mostly a carnivorous piscivore, it can be opportunistic and feed on plants, insects, worms, and crustaceans. Furthermore, it preys upon sick and injured fishes and scavenges on cadavers of fishes and other vertebrates (Pauly 1994). The popular man-eating reputation of piranhas is likely due to the necrophagous habits of these fish (Sazima and de Andrade Guimarães 1987), as only rarely have “feeding frenzy” attacks on large live prey have been observed. In the wild, red piranha exhibit social behavior and swim in schools, usually of 20–30 fish that feed together (Bellamy 1968; Sazima and Machado 1990). They engage in acoustic communication, which is sometimes exhibited along with aggressive behaviors (Millot et al. 2011). Red piranha mostly ambush fish from within the aquatic vegetation and dash after passing preys, and scan and pick the sediment while foraging on vegetation or invertebrates (Foxx 1972; Sazima and Machado 1990). They can endure long periods of prey shortage and starvation, in particular, when water levels drop and prey becomes scarce at the end of the summer.

Most fish in the wild are faced with short- to long-term fasting periods during their life, which can have profound behavioral and biochemical effects (Navarro and Gutiérrez 1995), and this phenomenon is more pronounced in carnivorous fish than in herbivorous or omnivorous fish (Navarro and Gutiérrez 1995). Carnivorous fish might be faced with the lack of adequate prey species in their environment more frequently, and need to invest more time and energy for successful capture, which presents a particular challenge after long periods of fasting.

Due to its unique biology and behaviors, the piranha represents a potentially valuable experimental model in feeding research that can provide important information on the effects of food restriction and feast/famine on feeding behavior. However, very few studies have examined how these animals can cope with starvation. Early metabolic studies show that, in red piranha, liver glycogen and lipids are mobilized rapidly during short-term starvation and low blood glucose levels induces food intake (Bellamy 1968). Studies have shown that fasting affects gene expression of a number of appetite-regulating hormones in the brain (e.g., orexin, CART) and in the intestine (e.g., PYY, leptin, ghrelin) of piranha (Volkoff 2014, 2015), suggesting that endocrine mechanisms might be in part responsible for the resistance of these fish to low food availability. Although these studies provide initial clues to the response of piranha to fasting, they only assess changes in expression of specific targeted genes. The availability of the *P. nattereri* genome allows us to investigate the genome-wide expression changes under conditions of fasting and to compare them to a satiated feeding regime, thus providing more broad context for understanding the physiological response of the fish to food restriction. We have assembled the piranha genome and used this resource to explore gene regulation in the brain following dietary restriction using high-throughput sequencing.

## Materials and Methods

### Genome Assembly and Annotation

The red piranha DNA used for sequencing was derived from a single female (Collection ID: Pna-1) among a maintained laboratory population. Using the predicted 1.6 Gb genome size estimate of the white piranha (Carvalho et al. 2002), total raw sequence coverage of Illumina reads was 76× (short overlapping reads, 3 kb, 8 kb, and 40 kb mate-paired libraries). The overlapping sequence reads (250 bp) were assembled using DiscoVar de novo (Weisenfeld et al. 2014) with default parameters. These reads are derived from a 400 bp library, thus making both pairs overlap in a region of ∼50 bp, a feature that is exploited by the assembler. This initial assembly of contigs was iteratively scaffolded with long paired reads (3 and 8 kb) using the program SSPACE (Boetzer et al. 2011) and gap filled with a version of Image (Tsai et al. 2010) modified for large genomes. All contigs and scaffolds were cleaned of contaminating sequences by performing a MegaBLAST (Zhang et al. 2000) of the contigs against adapter, bacterial and other vertebrate databases. The final assembly *P.nattereri* 1.0.2 was masked and then annotated for gene content using the NCBI pipeline described at http://www.ncbi.nlm.nih.gov/books/NBK169439/.

Repeats and transposable elements (TEs) were identified using RepeatModeler (version 1.0.11, http://www.repeatmasker.org/RepeatModeler/, default parameters). To screen the piranha genome for TEs, the resulting TE library was used as input for RepeatMasker (version 4.0, http://repeatmasker.org/). Characteristics of the repeat landscape (including element age and diversity) were plotted using the RepeatMasker perl script “createRepeatLandscape.”

### Positive Selection

To estimate genes under positive selection in piranha the protein and cDNA fasta files for several well-annotated species of fish representing the whole fish tree of life were downloaded from NCBI (supplementary table 1, Supplementary Material online). Orthologous proteins of all fish were identified using inparanoid (O’Brien et al. 2005)with default settings. For each gene with a protein ortholog across all species (comparison 1: *n* = 11,501, comparison 2: *n* = 3,949), the corresponding protein and cDNA sequences were aligned and converted into a codon alignment using pal2nal (version v14) (Suyama et al. 2006). Resulting sequences were aligned by MUSCLE (Edgar 2004) (option: -fastaout) and nonconserved blocks were removed using Gblocks (version 0.91 b) (Castresana 2000) (options: -b4 10 -b5 n –b3 5 –t = c). The Gblocks output was converted to paml format using an in-house script. Trees were built using Phylip (version 3.696, http://evolution.genetics.washington.edu/phylip.html) with *Danio rerio* (comparison 1) or *Latimeria chalumnae* (comparison 2) as outgroup (supplementary table 1, Supplementary Material online). For the generation of the phylogenetic tree, sequences from all orthologous genes of all fish (comparison 1: *n* = 11,501, comparison 2: *n* = 3,949) resulting from Gblocks were concatenated and aligned using MUSCLE. Distances were calculated using Phylip/dnadist (version 3.696, http://evolution.genetics.washington.edu/phylip.html) with *D.rerio* (comparison 1) or *L.chalumnae* (comparison 2) as outgroup (supplementary table 1, Supplementary Material online) using default settings. The tree was built by Phylip/neighbor using default settings. The phylogenetic tree was drawn from comparison 2 by the FigTree tool (http://tree.bio.ed.ac.uk/software/figtree/) with *L.chalumnae* as outgroup. For the phylogenetic analyses by maximum likelihood the “Environment for Tree Exploration” (ETE3) toolkit (Huerta-Cepas et al. 2016), which automates CodeML and Slr analyses by using preconfigured evolutionary models, was used. For the detection of genes under positive selection in piranha, we compared the branch-specific model bsA1 (neutral) with the model bsA (positive selection) using a likelihood ratio test (FDR ≤0.05). FDR was calculated using the “p.adjust” from the R package “stats.” To detect sites under positive selection, Naive Empirical Bayes (NEB) probabilities for all 4 classes were calculated for each site. Sites with a probability > 0.95 for either site class 2a (positive selection in marked branch and conserved in rest) or site class 2 b (positive selection in marked branch and relaxed in rest) were considered.

### Experimental Animals

Red-bellied piranhas (average weight 8.1 ± 0.1 g, average total length 6.8 ± 0.3 cm) were purchased from ABCee’s Aquatic Imports (Lasalle, QC, Canada) and kept in 65 L (61.0×33.0×33.0 cm^3^) glass aquaria under a simulated photoperiod of 16 h light:8 h dark, with constantly aerated and filtered water at 28 °C (two to five fish per tank). For starvation experiments, fish were placed in groups of 2–4 of size-matched fish in 5 (2 fed, 3 fasted) tanks to avoid cannibalism/predation on smaller fish. Fish were fed to satiety once a day (10:00 am), with flakes (41% protein, 12% fat, 2% fiber, 8.5% moisture, 8% ash, Omega Sea, Sitka, AK). Fish were acclimated under these standard conditions for 2 weeks before the start of the experiment. All fish were immature males or females (∼8 cm TL, piranhas mature at ∼16 cm TL, see Pauly 1994). For the fasting experiment, following the acclimation period, controls continued to be fed once a day and experimental fish were not fed for 10 days. On the sampling day, fed fish were fed at their regular feeding time and fed and fasted fish were sampled 30 minutes after feeding time.

Following experiments, fish were killed by immersion in 0.05% tricaine methanesulfonate (MS 222) (Syndel Laboratories, Vancouver, BC, Canada) followed by spinal section, and whole brain and intestines sampled. All fed fish had food in their gastrointestinal tracks (GIT), whereas fasted fish had empty GITs. Tissues were dissected and placed on ice in RNAlater (Qiagen, Mississauga, ON, Canada) and stored at −20 °C until RNA extractions were performed. All experiments were carried out in accordance with the principles published in the Canadian Council on Animal Care’s guide to the care and use of experimental animals (Memorial University protocol number 16-08-HV).

### Transcriptome Analysis

Total RNA was isolated with the RNAeasy kit (Qiagen) according to the company’s protocol. Total RNA was extracted from eyes, gills, spleen, kidney, ovary, liver, skin, heart, muscle, and brain to aid in gene annotation. For feeding trials, total RNA was isolated as before from the whole brains of three starved and four fed fish. We checked total RNA for quality on the Agilent Fragment Analyzer, then enriched for poly(A)+ RNA using the MicroPolyA Purist kit (Ambion, Carlsbad, CA). We used ScripSeq (Epicentre, Madison, WI) to generate strand-specific cDNA that was sequenced on the Illumina Hiseq2000 platform as 100 base paired-end reads (insert size of 400 bp). For transcriptome statistics, see supplementary table 2, Supplementary Material online. Sequences have been deposited at NCBI under BioProject ID PRJNA533530.

### Differential Gene Expression

All cDNA sequences from fed and fasted piranha were aligned to the *P.nattereri* 1.0.2 reference genome and read counts were calculated using the STAR aligner (Dobin et al. 2013) with default settings. Differentially expressed genes were detected using the Bioconductor package DESeq2 (Love et al. 2014). A gene was considered to be differentially expressed, if FDR ≤0.05 and baseMean ≥10. For the comparison of the intestine samples, a fold change > 4 in intestine was used since this is only preliminary data with one sample per treatment and no *P* value can be calculated. To discover enriched functional categories, human orthologs were determined by Ensembl biomart (http://www.ensembl.org/biomart/martview/0e0d4ed19703bfe45f84a3b9b452157a) and functionally clustered using the Database for Annotation, Visualization and Integrated Discovery (DAVID, https://david.ncifcrf.gov/home.jsp).

## Results and Discussion

### Assembly and Gene Annotation

We generated a draft assembly for a single female piranha (fig. 1) to use in studies of trait evolution unique to piranha. With a total sequenced genome input coverage of 76× (39× fragments, 26× 3 kb, and 11× 8 kb), we assembled a total of 1.25 Gb. The assembly is comprised of 283,518 scaffolds (including single contig scaffolds) with an N50 contig and scaffold length of 57 kb and 1.4 Mb, respectively. Despite a substantial scaffold number, remaining gap sequence size is estimated to be 33 Mb and overall assembly contiguity metrics are similar to fish genomes assembled with short-read approaches (supplementary table 3, Supplementary Material online). This suggests overall many assembly gaps are of small size and with future long-read approaches could be readily closed.

When masked with WindowMasker (Morgulis et al. 2006), we estimate 33.8% of the piranha genome contains interspersed highly repetitive elements, suggesting a shared overall genome structure when compared with the observed range of total repeats among other closely related fishes (supplementary table 4, Supplementary Material online). Using the NCBI annotation pipeline and RNA-seq transcript evidence from diverse tissue types, a total of 25,861 protein-coding genes, 7,838 noncoding, and 482 pseudogenes were predicted (supplementary table 4, Supplementary Material online). The Actinopterygii known proteins data set shows 85% average coverage (supplementary table 5, Supplementary Material online). The Actinopterygii set of proteins was used to make comparisons across species. Evaluation of the genome for completeness based on BUSCO (Assessing genome assembly and annotation completeness with Benchmarking Universal Single-Copy Orthologs) identified 95.3% complete genes from the 4,584 Actinopterygii data set (supplementary table 6, Supplementary Material online). In summary, our various measures of gene representation suggest we have assembled most of the protein-coding genes in the red piranha genome. A full report of the gene annotation can be found at https://www.ncbi.nlm.nih.gov/genome/annotation_euk/Pygocentrus_nattereri/100/

**Fig. 2.**
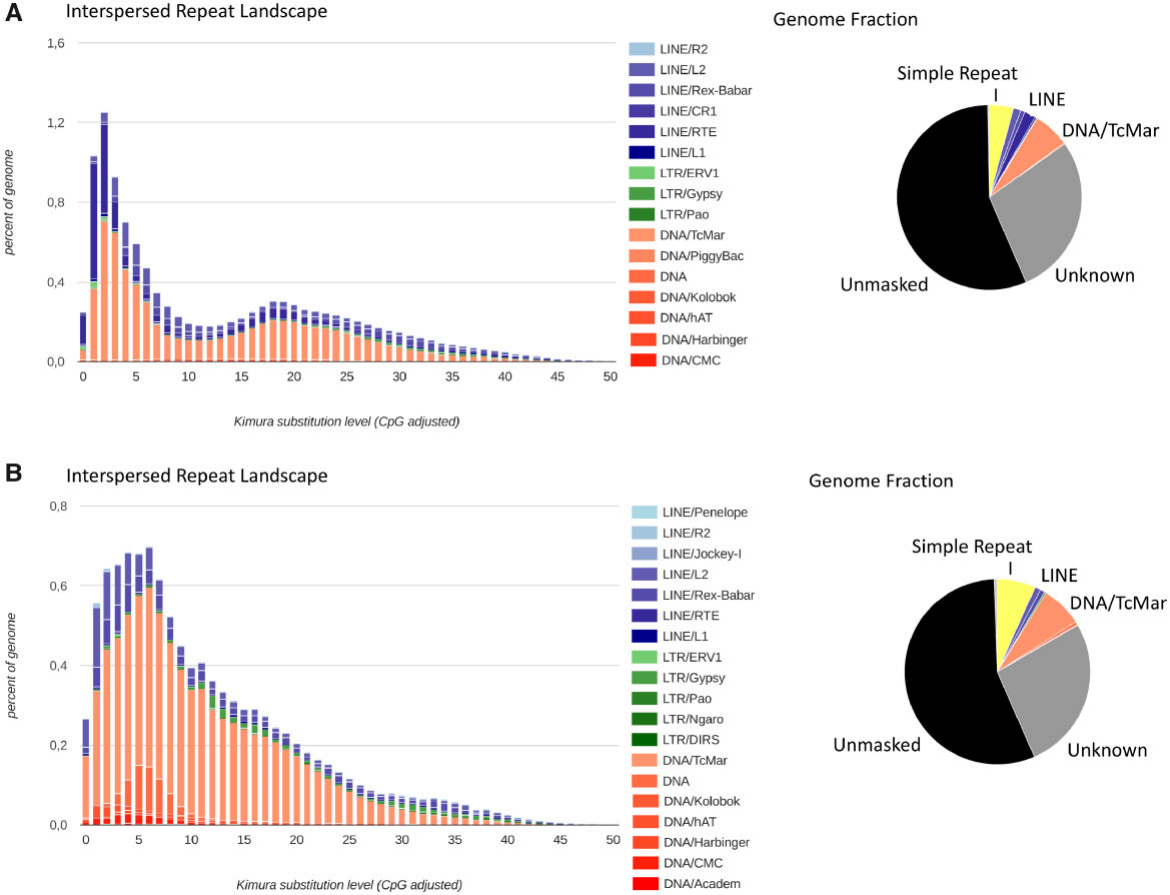
—Interspersed repeat landscape, revealing the copy-divergence analysis of TE classes, based on Kimura distances. Percentages of TEs in genomes (*Y*-axis) are clustered based on their Kimura values (*X*-axis; *K* values from 0 to 50; arbitrary values). Older copies are located on the right side of the graphs while rather recent copies are located on the left side. (*A*) Piranha and (*B*) Cavefish. Pie charts show the fraction of the genomes covered by known and unknown repeats.

The RepeatModeller output for the piranha genome masked ∼44% (supplementary table 7, Supplementary Material online), which is in the range of most sequenced bony fish genomes (Chalopin et al. 2015). TEs constitute the majority of the known repeat content (40%), which is in the same range as the blind cave fish, another representative of the Characidae with a similar landscape of TEs (supplementary table 8, Supplementary Material online and fig. 2). The majority of the classified TE’s are LINEs (4.3%) and DNA elements (6.7%), 28.3% of the genome being unclassified TEs.

The transposon history analysis of classified TE’s revealed mainly a recent burst (fig. 2), mostly of LINE elements, while another more ancient expansion of the mobilome seen in many other teleost genomes (e.g., platyfish, tilapia; Brawand 2014; Chalopin et al. 2015) is not evident in the piranha genome. In comparison to the cavefish, which also lacks the ancient expansion signature, the more recent wave of TE propagation is even younger.

### Gene and Genome Evolution

**Fig. 3.**
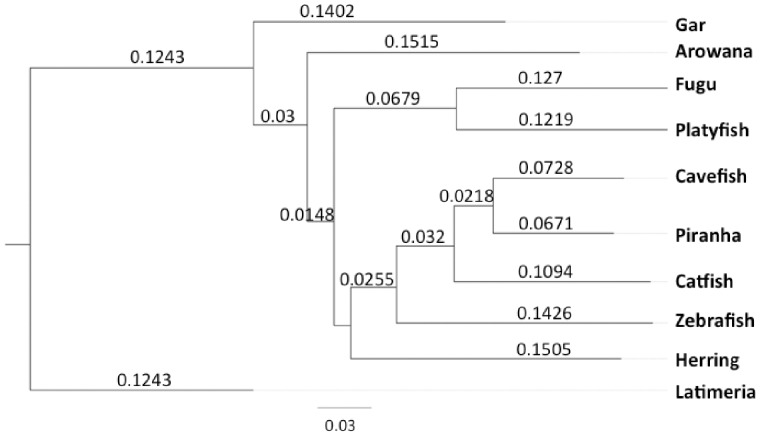
—Phylogenetic tree from the orthology gene set. Numbers on the branches indicate branch lengths. Bar represents 0.03 substitutions per site.

The availability of a whole-genome sequence offers the opportunity to search for genetic factors that are involved in the evolution of specific traits and adaptation of piranhas. We analyzed the predicted gene set for evidence of positive selection in piranha. Only sequences that aligned unambiguously in all of the selected species were considered for selection analysis. We produced two comparisons, one that includes only the closest related species from which high quality genomes are available, the cavefish *Astyanax mexicanus* and the channel catfish, *Ictalurus punctatus*, with the zebrafish as outgroup (comparison 1). Comparison 2 was made between nine actinopterygian fish species with the coelacanth genome as outgroup (fig. 3).

This analysis identified 44 genes in comparison 1 and 164 genes in comparison 2 (supplementary table 9, Supplementary Material online) with evidence of piranha-specific positive selection. A GO-term analysis (supplementary fig. 1, Supplementary Material online) revealed that positively selected genes were enriched for GO terms from metabolism and mitochondrial processes. In addition, epidermal growth factor signaling related GO terms show enrichment. The list of genes under positive selection in the piranha contains many proteins involved in structure and function of the nervous system, for example, glutamate and purinergic receptors, neurexophilin-related protein, slit guidance ligand 1, NDGR4, trace amine-associated receptor 1, cerebellar degeneration-related protein 2, and glial fibrillary acidic protein. Transient receptor potential cation channel M5 plays a role in the perception of taste (Yoshida et al. 2007) and glutamate exchanger xCT is known to mediate resilience to stress (Nasca et al. 2017). This all may be related to the well-developed sensorimotor system of piranha as a fast swimmer and prey detector.

Given the ability of piranha to cope with long periods of starvation several other genes under positive selection are noteworthy. As important regulator of fasting metabolism, adenylate kinase 4 (*ak4*) plays a key role in cellular energy homeostasis and neuropeptide FF receptor is related to growth and body weight regulation (Peng et al. 2016).

### Comparative Expression Profile Analysis of Starved and Fed Piranhas

Piranhas are adapted to long periods of prey shortage, in particular, when water levels get low and prey becomes scarce at the end of the summer. Changes in metabolism occur during food deprivation. During fasting, the mobilization of energy reserves occurs in several tissues, and it tends to be sequential, with carbohydrates utilized first, followed by fat and then protein (Woo and Fung 1981; Barcellos et al. 2010; Bar and Volkoff 2012). In some species, lipid storage primarily occurs in the liver, whereas in other species, they may predominantly be stored in muscles and in mesentery, as seen in most piranhas (Hiane et al. 2002; Ferreira 2010). A mutation in the insulin receptor, which leads to dysregulation of blood glucose levels, has allowed the blind cavefish, *A.mexicanus*, also like the piranhas a member of the Characiformes, to adapt to prolonged periods of starvation (Riddle et al. 2018).

With the availability of a high-quality reference genome and the prediction of the *P. nattereri* protein coding genes, it is now possible to perform genome wide transcriptomic studies that were lacking so far. We could for the first time estimate genome-wide expression changes in the whole brain under extreme conditions of fasting and compare to conditions of sufficient feeding regime. To control for the efficiency of our experimental conditions, RNA-seq on one intestine from each group was conducted. Changes in digestive enzymes and “classical” gut hormones, and gene regulations that are indicative of structural reorganization of the gut under fasting conditions (see supplementary table 10, Supplementary Material online) confirmed that the fish under starvation conditions were indeed under nourishment stress.

In brain of food-deprived fish, the metabolic consequences of starvation have been studied only in few species so far, mostly by measuring metabolites and glucose sensors in salmonids (Soengas and Aldegunde 2002). Two studies using targeted qPCRs (Rimoldi et al. 2016) or zebrafish microarrays (Drew et al. 2008) have also been conducted. However, comparable information from piranha is missing so far (Polakof et al. 2012). The availability of a high quality piranha reference genome allowed us to perform the first RNA-seq analysis of brains from starved fish. In the piranha whole brain, starvation had significant effects on the regulated transcriptome. We found 557 transcripts to be downregulated and 394 transcripts to be upregulated in starved fish (logFC ranging from −4.8 to 6.3; supplementary table 10 and figs. 2 and 3, Supplementary Material online). It is likely that even more striking effects may be noticeable after longer periods of fasting. However, we avoided increasing the fasting periods as this would be expected to increase in-tank cannibalism. The differentially expressed genes were enriched for several functional categories, for example, lipid metabolism, metabolic signaling, reorganization of extracellular matrix and proliferation and growth (supplementary fig. 4, Supplementary Material online). By parsing the differentially expressed genes with known role in metabolism, we find genic starvation responses in lipid transport (e.g., *nrf6*), including many genes of the fatty acid (FA) metabolism, genes (e.g., *sterol regulatory element binding transcription factor 1*, *fatty acid synthase*, *desaturase 2*, *elongase 5*). Up to 20% of the total brain’s energy is provided by mitochondrial oxidation of FAs in mammals (Panov et al. 2014) supporting our observation that in the fasted piranha brain a shift toward transcriptome programs involved in brain energy regulation is observed. FA availability in the brain appears to be very important to the regulation of energy balance, as infusions of FAs into the brain inhibit food intake, although paradoxically, plasma FAs are highest during fasting (Wang and Eckel 2012). Fasting-induced adipose factor/angiopoietin-related protein 4 (*angptl4*) was strongly upregulated in the brain of starved piranhas. Overexpression of this gene has been shown in mice to be a mediator or starvation response acting mainly on lipid metabolism (Mandard et al. 2006).

Glucose and its derivative lactate, as well as ketone bodies (following a long fast) are the main energy substrate for the brain of mammals (Panov et al. 2014) and fish (Soengas and Aldegunde 2002). Genes involved in the metabolism of carbohydrates and lactate (e.g., *pyruvate dehyogenase kinase*, *facilitated glucose transporter 1*) and genes involved in ketone body formation (e.g., *acetyl CoA-acyltransferase 2*) were significantly affected by fasting. Brain oxidation of ketone bodies is known to increase during food deprivation in some fish (Soengas and Aldegunde 2002).

Changes in metabolic parameters are often accompanied by changes in feeding-regulating hormones produced by both brain and peripheral tissues. These hormones affect feeding centers in the brain to either stimulate (orexigenic) or inhibit (anorexigenic) feeding (Volkoff 2011, 2016; Ronnestad et al. 2017) Short-term fasting usually increases the expression of orexigenic factors and decreases that of anorexigenic factors. This has been shown in piranha (Volkoff 2014, 2015) and other characiform fish such as the large fruit-eating pacu, a close relative to the piranhas (Volkoff et al. 2017), dourado (Volkoff 2016), and cavefish (Wall and Volkoff 2013). Our data uncovered only small and nonsignificant changes in the expression of endocrine factors regulating appetite, such as CART and leptin B.

As a consequence of starvation several genes of neurotransmitter metabolisms (e.g., *tryptophane 5-hydroxylase*, *monoxygenase DBH-like*, *GABA transporter 2*, *serotonin carrier slc7a2* aka *cat2*) and neurogenesis (e.g., *neural cell adhesion 1*, *neurabin 2*, *synaptotagmin 3*, *glial fibrillary acidic protein*) were regulated. An impact was also noted on so-called “immediate-early” genes that are known to play a role in neural plasticity (Mukherjee et al. 2018) including *early growth response protein2* and members of the *fos* transcription factor family, which all were downregulated. In addition, delta opioid receptor Oprd1a, a component of the reward system (Pradhan et al. 2011) was downregulated, probably because satiety as initiator of a reward response is not reached any more under starvation conditions.

In search for prey, hungry piranhas need to exhibit aggression-boldness behavior. A susceptibility gene for anxiety, *egr2* (Mcgregor 2013), is downregulated in the brains of starved piranhas, which may contribute to increased boldness to aid the acquisition of food. We also noted significant upregulation under food deprivation of components of FGF signaling (*fgf 9*, *fgf 8*, *fgf 1*) a major pathway known to regulate aggression-boldness in zebrafish (Norton et al. 2011).

## Conclusion

Piranhas are known for a unique combination of morphological, physiological and behavioral traits, which makes them top freshwater predators in their habitats. The analyses of our whole-genome assembly and annotation revealed signatures of positive selection in several genes related to neurotransmission, behavior, and metabolism regulation that are candidates for being involved in adaptations to the specific feeding lifestyle of these fishes. Most data on fasting-induced metabolic changes in fish pertain to liver and muscle tissue (e.g., carp; Wang et al. 2006; Gong et al. 2017; rainbow trout; Salem et al. 2007; and zebrafish; Drew et al. 2008). The brain is also a major organ involved in the regulation of feeding (Ronnestad et al. 2017). This transcriptomic study is the first of its kind in piranha and provides new information on changes in the genome under caloric restriction. In particular, it provides evidence for the upregulation of genes involved in metabolism, suggesting an increased utilization of storage fuels and a brain energy sparring outcome in the piranha is consistent with that seen in other fish species. In summary, we find gene candidates for the complex process of feeding and starvation regulation, and to the more aggressive behavior of starved piranha. Future studies will also reveal how the teleost specific genome duplication and the presence of two genes (as opposed to a single gene in tetrapods) involved in processes of metabolism regulation and behavior might have impacts on the regulation of starvation and metabolism in general. The availability of a reference genome for the whole species group of piranhas will provide the basis for a better understanding of the biology and evolution of these signature fish of the large South American river basins.

## Supplementary Material

Supplementary data are available at *Genome Biology and Evolution* online.

## Supplementary Material

Supplementary_Data_evz139

## Notes

**Data deposition:** Sequences have been deposited at NCBI under BioProject ID PRJNA533530.

## References

[evz139-B1] Bar N, Volkoff H. 2012 Comparative physiology of fasting, starvation, and food limitation. In: McCue MD, editor. Adaptation of the physiological, endocrine, and digestive system functions to prolonged food deprivation in fish Berlin, Heidelberg: Springer Berlin Heidelberg p. 69–89.

[evz139-B2] Barcellos LJG, Marqueze A, Trapp M, Quevedo RM, Ferreira D. 2010 The effects of fasting on cortisol, blood glucose and liver and muscle glycogen in adult jundiá *Rhamdia quelen*. Aquaculture 300(1–4):231–236.

[evz139-B3] Bellamy D. 1968 Metabolism of the red piranha (*Rooseveltiella nattereri*) in relation to feeding behaviour. Comp Biochem Physiol. 25(1):343–347. 5657211 10.1016/0010-406x(68)90942-0

[evz139-B4] Boetzer M, Henkel CV, Jansen HJ, Butler D, Pirovano W. 2011 Scaffolding pre-assembled contigs using SSPACE. Bioinformatics 27(4):578–579. 21149342 10.1093/bioinformatics/btq683

[evz139-B5] Brawand D, et al 2014 The genomic substrate for adaptive radiation in African cichlid fish. Nature 513(7518):375–381. 25186727 10.1038/nature13726 PMC4353498

[evz139-B6] Carvalho ML, Oliveira C, Navarrete MC, Froehlich O, Foresti F. 2002 Nuclear DNA content determination in Characiformes fish (Teleostei, Ostariophysi) from the Neotropical region. Genet Mol Biol. 25(1):49–55.

[evz139-B7] Castresana J. 2000 Selection of conserved blocks from multiple alignments for their use in phylogenetic analysis. Mol Biol Evol. 17(4):540–552. 10742046 10.1093/oxfordjournals.molbev.a026334

[evz139-B8] Chalopin D, Naville M, Plard F, Galiana D, Volff JN. 2015 Comparative analysis of transposable elements highlights mobilome diversity and evolution in vertebrates. Genome Biol Evol. 7(2):567–580. 25577199 10.1093/gbe/evv005 PMC4350176

[evz139-B9] Dobin A, et al 2013 STAR: ultrafast universal RNA-seq aligner. Bioinformatics 29(1):15–21. 23104886 10.1093/bioinformatics/bts635 PMC3530905

[evz139-B10] Drew RE, et al 2008 Effect of starvation on transcriptomes of brain and liver in adult female zebrafish (*Danio rerio*). Physiol Genomics. 35(3):283–295. 18728227 10.1152/physiolgenomics.90213.2008 PMC2585019

[evz139-B11] Edgar RC. 2004 MUSCLE: multiple sequence alignment with high accuracy and high throughput. Nucleic Acids Res. 32(5):1792–1797. 15034147 10.1093/nar/gkh340 PMC390337

[evz139-B12] Ferreira AA. 2010 Peixe piranha (*Pygocentrus nattereri*) do pantanal: composição em ácidos graxos e mudanças com o processamento e estocagem [master thesis]. [Campo Grande (MS, Brazil)]: Universidade Federal do Mato Grosso do Sul.

[evz139-B13] Foxx RM. 1972 Attack preferences of the red-bellied piranha (*Serrasalmus nattereri*). Anim Behav. 20(2):280–283.

[evz139-B14] Gong Y, et al 2017 Effects of food restriction on growth, body composition and gene expression related in regulation of lipid metabolism and food intake in grass carp. Aquaculture 469:28–35.

[evz139-B15] Grubich JR, Huskey S, Crofts S, Orti G, Porto J. 2012 Mega-Bites: extreme jaw forces of living and extinct piranhas (Serrasalmidae). Sci Rep. 2(1):1009. 23259047 10.1038/srep01009 PMC3526859

[evz139-B16] Hiane PA, Leal Filho AF, Ramos Filho MM, Ramos MIL. 2002 Teores de colesterol e lipídios totais em seis espécies de peixes capturados na região pantaneira do Estado Mato Grosso do Sul. Bol Centro Pesqui Process Aliment. 20:65–74.

[evz139-B17] Huerta-Cepas J, Serra F, Bork P. 2016 ETE 3: reconstruction, analysis, and visualization of phylogenomic data. Mol Biol Evol. 33(6):1635–1638. 26921390 10.1093/molbev/msw046 PMC4868116

[evz139-B18] Love MI, Huber W, Anders S. 2014 Moderated estimation of fold change and dispersion for RNA-seq data with DESeq2. Genome Biol. 15(12):550. 25516281 10.1186/s13059-014-0550-8 PMC4302049

[evz139-B19] Mandard S, et al 2006 The fasting-induced adipose factor/angiopoietin-like protein 4 is physically associated with lipoproteins and governs plasma lipid levels and adiposity. J Biol Chem. 281(2):934–944. 16272564 10.1074/jbc.M506519200

[evz139-B20] Mcgregor NW. 2013. The identification of novel susceptibility genes involved in anxiety disorders. Stellenbosch, South Africa: Stellenbosch University.

[evz139-B21] Millot S, Vandewalle P, Parmentier E. 2011 Sound production in red-bellied piranhas (*Pygocentrus nattereri*, Kner): an acoustical, behavioural and morphofunctional study. J Exp Biol. 214(Pt 21):3613–3618. 21993790 10.1242/jeb.061218

[evz139-B22] Morgulis A, Gertz EM, Schaffer AA, Agarwala R. 2006 WindowMasker: window-based masker for sequenced genomes. Bioinformatics 22(2):134–141. 16287941 10.1093/bioinformatics/bti774

[evz139-B23] Mukherjee D, et al 2018 Salient experiences are represented by unique transcriptional signatures in the mouse brain. eLife 7:e31220. 29412137 10.7554/eLife.31220 PMC5862526

[evz139-B24] Nasca C, et al 2017 Role of the astroglial glutamate exchanger xCT in ventral hippocampus in resilience to stress. Neuron 96(2):402–413. 29024663 10.1016/j.neuron.2017.09.020

[evz139-B25] Navarro I, Gutiérrez J. 1995 Biochemistry and molecular biology of fishes. In: Hochachka PW, Mommsen TP, editors. Chapter 17 Fasting and starvation Amsterdam, The Netherlands: Elsevier p. 393–434.

[evz139-B26] Norton WH, et al 2011 Modulation of Fgfr1a signaling in zebrafish reveals a genetic basis for the aggression-boldness syndrome. J Neurosci. 31(39):13796–13807. 21957242 10.1523/JNEUROSCI.2892-11.2011 PMC6633160

[evz139-B27] O’Brien KP, Remm M, Sonnhammer ELL. 2005 Inparanoid: a comprehensive database of eukaryotic orthologs. Nucleic Acids Res. 33:D476–D480. 15608241 10.1093/nar/gki107 PMC540061

[evz139-B28] Panov A, Orynbayeva Z, Vavilin V, Lyakhovich V. 2014 Fatty acids in energy metabolism of the central nervous system. BioMed Res Int. 2014:1. 10.1155/2014/472459 PMC4026875 24883315

[evz139-B29] Pauly D. 1994 Quantitative analysis of published data on the growth, metabolism, food consumption, and related features of the red-bellied piranha, *Serrasalmus nattereri* (Characidae). Environ Biol Fish. 41(1–4):423–437.

[evz139-B30] Peng W, et al 2016 An ultra-high density linkage map and QTL mapping for sex and growth-related traits of common carp (*Cyprinus carpio*). Sci Rep. 6(1):26693. 27225429 10.1038/srep26693 PMC4880943

[evz139-B31] Polakof S, Panserat S, Soengas JL, Moon TW. 2012 Glucose metabolism in fish: a review. J Comp Physiol B. 182(8):1015–1045. 22476584 10.1007/s00360-012-0658-7

[evz139-B32] Pradhan AA, Befort K, Nozaki C, Gavériaux-Ruff C, Kieffer BL. 2011 The delta opioid receptor: an evolving target for the treatment of brain disorders. Trends Pharmacol Sci. 32(10):581–590. 21925742 10.1016/j.tips.2011.06.008 PMC3197801

[evz139-B33] Riddle MR, et al 2018 Insulin resistance in cavefish as an adaptation to a nutrient-limited environment. Nature 555(7698):647–651. 29562229 10.1038/nature26136 PMC5989729

[evz139-B34] Rimoldi S, Benedito-Palos L, Terova G, Perez SJ. 2016 Wide-targeted gene expression infers tissue-specific molecular signatures of lipid metabolism in fed and fasted fish. Rev Fish Biol Fisheries. 26(1):93–108.

[evz139-B35] Ronnestad I, et al 2017 Appetite-controlling endocrine systems in teleosts. Front Endocrinol. 8:73. 10.3389/fendo.2017.00073 PMC5394176 28458653

[evz139-B36] Salem M, Silverstein J, Rexroad CE, Yao J. 2007 Effect of starvation on global gene expression and proteolysis in rainbow trout (*Oncorhynchus mykiss*). BMC Genomics 8(1):328. 17880706 10.1186/1471-2164-8-328 PMC2040161

[evz139-B37] Sazima I, de Andrade Guimarães S. 1987 Scavenging on human corpses as a source for stories about man-eating piranhas. Environ Biol Fish. 20(1):75–77.

[evz139-B38] Sazima I, Machado FA. 1990 Underwater observations of piranhas in western Brazil. Environ Biol Fish. 28(1–4):17–31.

[evz139-B39] Soengas JL, Aldegunde M. 2002 Energy metabolism of fish brain. Comp Biochem Physiol B Biochem Mol Biol. 131(3):271–296. 11959012 10.1016/s1096-4959(02)00022-2

[evz139-B40] Suyama M, Torrents D, Bork P. 2006 PAL2NAL: robust conversion of protein sequence alignments into the corresponding codon alignments. Nucleic Acids Res. 34(Web Server):W609–W612. 16845082 10.1093/nar/gkl315 PMC1538804

[evz139-B41] Tsai IJ, Otto TD, Berriman M. 2010 Improving draft assemblies by iterative mapping and assembly of short reads to eliminate gaps. Genome Biol. 11(4):R41. 20388197 10.1186/gb-2010-11-4-r41 PMC2884544

[evz139-B42] Volkoff H. 2011 Encyclopedia of fish physiology. In: Farrell AP, editor. Hormonal control of reproduction and growth | control of appetite in fish San Diego: Academic Press p. 1509–1514.

[evz139-B43] Volkoff H. 2014 Appetite regulating peptides in red-bellied piranha, *Pygocentrus nattereri*: cloning, tissue distribution and effect of fasting on mRNA expression levels. Peptides 56:116–124. 24721336 10.1016/j.peptides.2014.03.022

[evz139-B44] Volkoff H. 2015 Cloning, tissue distribution and effects of fasting on mRNA expression levels of leptin and ghrelin in red-bellied piranha (*Pygocentrus nattereri*). Gen Comp Endocrinol. 217–218:20–27. 10.1016/j.ygcen.2015.05.004 25980684

[evz139-B45] Volkoff H. 2016 The neuroendocrine regulation of food intake in fish: a review of current knowledge. Front Neurosci. 10:540. 27965528 10.3389/fnins.2016.00540 PMC5126056

[evz139-B46] Volkoff H, Estevan Sabioni R, Coutinho LL, Cyrino JEP. 2017 Appetite regulating factors in pacu (*Piaractus mesopotamicus*): tissue distribution and effects of food quantity and quality on gene expression. Comp Biochem Physiol A Mol Integr Physiol. 203:241–254. 27717774 10.1016/j.cbpa.2016.09.022

[evz139-B47] Wall A, Volkoff H. 2013 Effects of fasting and feeding on the brain mRNA expressions of orexin, tyrosine hydroxylase (TH), PYY and CCK in the Mexican blind cavefish (*Astyanax fasciatus mexicanus*). Gen Comp Endocrinol. 183:44–52. 23305930 10.1016/j.ygcen.2012.12.011

[evz139-B48] Wang H, Eckel RH. 2012 Lipoprotein lipase in the brain and nervous system. Annu Rev Nutr. 32(1):147–160. 22540257 10.1146/annurev-nutr-071811-150703 PMC4065112

[evz139-B49] Wang T, Hung CCY, Randall DJ. 2006 The comparative physiology of food deprivation: from feast to famine. Annu Rev Physiol. 68(1):223–251. 16460272 10.1146/annurev.physiol.68.040104.105739

[evz139-B50] Weisenfeld N, et al 2014 Comprehensive variation discovery in single human genomes. Nat Genet. 46(12):1350–1355. 25326702 10.1038/ng.3121 PMC4244235

[evz139-B51] Woo NYS, Fung ACY. 1981 Studies on the biology of the red sea bream *Chrysophrys major*—IV. Metabolic effects of starvation at low temperature. Comp Biochem Physiol A Physiol. 69(3):461–465.

[evz139-B52] Yoshida Y, et al 2007 Transient receptor potential channel M5 and phospholipaseC-beta2 colocalizing in zebrafish taste receptor cells. Neuroreport 18(15):1517–1520. 17885593 10.1097/WNR.0b013e3282ec6874

[evz139-B53] Zhang Z, Schwartz S, Wagner L, Miller W. 2000 A greedy algorithm for aligning DNA sequences. J Comput Biol. 7(1–2):203–214. 10890397 10.1089/10665270050081478

