# Supplementary material for: The Piranha Genome Provides Molecular Insight Associated to Its Unique Feeding Behavior
Source: Genome Biol Evol. 2019 Jul 8;11(8):2099–106. doi: 10.1093/gbe/evz139 (PMC6681833; doi:10.1093/gbe/evz139)
Supplement: Supplementary_Data_evz139 [file supplementary_data_evz139.zip › Suppl legends.docx]

**Supplementary Fig. 1**: Barplots of enriched functional categories of positively selected genes for comparison1 (A), comparison2 (B) and enriched GO-terms 'biological process' (C) and 'cellular component' (D) for comparison2. Blue bars indicate the expected number of genes within a category given the result to be random and red bars the observed number of genes within a category in the selected gene set. Enriched categories were selected using the online tool DAVID with default settings. For the functional annotation human orthologes of piranha genes were used. Significance of categories is decreasing from bottom to top.

**Supplementary Fig. 2**: 3D scatterplot of the correspondence analysis of fed (black) and starved (grey) piranha brain samples. The x, y and z axes represent component 1, 2 and 3. Samples are clearly separated on the main axis (x).

**Supplementary Fig. 3:** Heatmap of 951 genes differentially expressed between the two feeding regimes (adj. p-value <=0.05 and baseMeann >=10). Columns represent individual samples (x-axis), while rows represent differentially expressed genes (y-axis). Heatmap color displays the z-score ranging from blue (z-score of **−**2) to yellow (z-score of +2). The relative expression for each gene is represented by color intensity, with blue indicating low expression and yellow indicating high expression levels.

**Supplementary Fig. 4**: Barplots of enriched functional categories of differentially expressed genes between the two feeding regimes. Blue bars indicate the expected number of genes within a category given the result to be random and red bars the observed number of genes within a category in the selected gene set. Enriched categories were selected using the online tool DAVID with default settings. For the functional annotation human orthologs of piranha genes were used. Significance of categories is decreasing from bottom to top.
