# Supplementary material for: The Piranha Genome Provides Molecular Insight Associated to Its Unique Feeding Behavior
Source: Genome Biol Evol. 2019 Jul 8;11(8):2099–106. doi: 10.1093/gbe/evz139 (PMC6681833; doi:10.1093/gbe/evz139)
Supplement: Supplementary_Data_evz139 [file supplementary_data_evz139.zip › Supplementary Tables 1-8.docx]

**Supplementary Tables**

**Supplementary table 1**: Genomes used for phylogenetic analyses

|  | comparison.1 | comparison.2 | genome release |
| --- | --- | --- | --- |
| *Pygocentrus nattereri* | + | + | 100 |
| *Astyanax mexicanus* | + | + | 102 |
| *Ictalurus punctatus* | + | + | 100 |
| *Danio rerio* | outgroup | + | 106 |
| *Clupea harengus* |  | + | 100 |
| *Scleropages formosus* |  | + | 100 |
| *Lepisosteus oculatus* |  | + | 101 |
| *Xiphophorus maculatus* |  | + | 101 |
| *Takifugu rubripes* |  | + | 101 |
| *Latimeria chalumnae* |  | outgroup | 101 |

**Supplementary table 2**: Transcriptome statistics

|  | Number of input reads | Uniquely mapped reads % |
| --- | --- | --- |
| brain fed1 | 70077098 | 84,60% |
| brain fed2 | 65164644 | 81,60% |
| brain fed3 | 80139560 | 83,07% |
| brain fed4 | 77645354 | 84,26% |
| brain fed5 | 76298364 | 81,53% |
| brain starved1 | 75891476 | 82,48% |
| brain starved2 | 65879694 | 83,87% |
| brain starved3 | 70716042 | 80,79% |
| brain starved4 | 77846748 | 81,35% |
| intestine fed | 71446410 | 82,44% |
| intestine starved | 81450912 | 79,68% |

**Supplementary table 3:** Representative assembly metrics for sequenced fish genomes

| Common name | Assembled version | N50 contig (kb) | N50 scaffold (Mb) | Total assembly size (Gb) |
| --- | --- | --- | --- | --- |
| Red piranha | Pygocentrus nattereri 1.0.2 | 57 | 1.4 | 1.25 |
| Blind cavefish | Aystyanax mexicanus 1.0.2 | 15 | 1.7 | 0.95 |
| Duck-billed golden line | SAMN03320099.wgs v1.1 | 17 | 1.2 | 1.51 |
| Channel catfish | IpCoco 1.2 | 77 | 7.7 | 0.77 |

All species-specific assembly metrics derived from the NCBI assembly archive. Estimates use all contigs and scaffolds >200bp.

**Supplementary table 4:** Comparative gene annotation measures for sequenced fish genomes

| Common name | Assembled version | Protein coding genes | Total ncRNA | mRNAs | Repeats |
| --- | --- | --- | --- | --- | --- |
| Red piranha | *Pygocentrus nattereri* 1.0.2 | 25,861 | 7,838 | 44,351 | 33.8 |
| Blind cavefish | *Aystyanax mexicanus* 1.0.2 | 23,628 | 1,062 | 33,353 | 40.9 |
| Duck-billed golden line | SAMN03320099.wgs v1.1 | 42,840 | 5,152 | 68,461 | 36.3 |
| Channel catfish | IpCoco 1.2 | 23,218 | 4,177 | 47,961 | 33.1 |

All species-specific annotation metrics derived from the NCBI annotation archive.

**Supplementary table 5**: Estimates of gene and protein representation for sequenced fish genomes.

|  |  | Transcript^1^ | | Protein^2^ | |
| --- | --- | --- | --- | --- | --- |
| Common name | Assembled version | Average % identity | Average % coverage | Average % identity | Average % coverage |
| Red piranha | *Pygocentrus nattereri* 1.0.2 | 99.4 | 94.6 | 69 | 84.8 |
| Blind cavefish | *Aystyanax mexicanus* 1.0.2 | 99.2 | 98.8 | 69.5 | 75.5 |
| Duck-billed golden line | SAMN03320099.wgs v1.1 | NA | NA | 72.2 | 80.5 |
| Channel catfish | IpCoco 1.2 | 98.9 | 93.3 | 69.4 | 78.4 |

^1^ Transcript alignments to same species GenBank transcripts.

^2^ All protein alignments were to the Actinopterygii GenBank proteins known (n=75,619).

**Supplementary table 6:** BUSCO scores for the completeness of the piranha genome annotation (<http://busco.ezlab.org>; database actinopterygii_odb9, reference species zebrafish)

|  | Actinopterygii Gene Set | % |
| --- | --- | --- |
| Complete BUSCOs (C) | 4,371 | 95.3 |
| Complete and single-copy BUSCOs (S) | 4,177 | 91.1 |
| Complete and duplicated BUSCOs (D) | 194 | 4.2 |
| Fragmented BUSCOs (F) | 67 | 1.5 |
| Missing BUSCOs (M) | 146 | 3.2 |
| Total BUSCO groups searched | 4,584 |  |

**Supplementary table 7**: Repeat and transposable element statistics in the Piranha genome

Repeat Modeller output: bases masked: 563,194,035 bp (43.82 %)

|  | number of elements* | | length occupied | percentage of sequence |
| --- | --- | --- | --- | --- |
| SINEs: | 0 | 0 bp | | 0.00 % |
| ALUs | 0 | 0 bp | | 0.00 % |
| MIRs | 0 | 0 bp | | 0.00 % |
| LINEs: | 149153 | 55059694 bp | | 4.28 % |
| LINE1 | 7331 | 3079981 bp | | 0.24 % |
| LINE2 | 55417 | 16560840 bp | | 1.29 % |
| L3/CR1 | 315 | 46677 bp | | 0.00 % |
| LTR elements: | 8411 | 4726525 bp | | 0.37 % |
| ERVL | 0 | 0 bp | | 0.00 % |
| ERVL-MaLRs | 0 | 0 bp | | 0.00 % |
| ERV_classI | 3319 | 1375328 bp | | 0.11 % |
| ERV_classII | 0 | 0 bp | | 0.00 % |
| DNA elements: | 280210 | 85760496 bp | | 6.67 % |
| hAT-Charlie | 2442 | 533328 bp | | 0.04 % |
| TcMar-Tigger | 0 | 0 bp | | 0.00 % |
| Unclassified: | 2287096 | 363769441 bp | | 28.30 % |
| Total interspersed repeats: |  | 509316156 bp | | 39.63 % |
| Small RNA: | 0 | 0 bp | | 0.00 % |
| Satellites: | 0 | 0 bp | | 0.00 % |
| Simple repeats: | 669664 | 48034258 bp | | 3.74 % |
| Low complexity: | 61582 | 6340039 bp | | 0.49 % |

*most repeats fragmented by insertions or deletions have been counted as one element

**Supplementary table 8**: Repeat and transposable element statistics in the cavefish genome

Repeat Modeller output: bases masked: 588,746,568 bp (44.09 %)

|  | number of elements* | | length occupied | percentage of sequence |
| --- | --- | --- | --- | --- |
| SINEs: | 0 | 0 bp | | 0.00 % |
| ALUs | 0 | 0 bp | | 0.00 % |
| MIRs | 0 | 0 bp | | 0.00 % |
| LINEs: | 63911 | 26870955 bp | | 2.01 % |
| LINE1 | 2336 | 1070435 bp | | 0.08 % |
| LINE2 | 38124 | 13532800 bp | | 1.01 % |
| L3/CR1 | 0 | 0 bp | | 0.00 % |
| LTR elements: | 5708 | 6630702 bp | | 0.50 % |
| ERVL | 0 | 0 bp | | 0.00 % |
| ERVL-MaLRs | 0 | 0 bp | | 0.00 % |
| ERV_classI | 491 | 324565 bp | | 0.02 % |
| ERV_classII | 0 | 0 bp | | 0.00 % |
| DNA elements: | 469341 | 108499514 bp | | 8.13 % |
| hAT-Charlie | 2993 | 578192 bp | | 0.04 % |
| TcMar-Tigger | 0 | 0 bp | | 0.00 % |
| Unclassified: | 1779956 | 358586509 bp | | 26.86 % |
| Total interspersed repeats: |  | 500587680 bp | | 37.49 % |
| Small RNA: | 0 | 0 bp | | 0.00 % |
| Satellites: | 0 | 0 bp | | 0.00 % |
| Simple repeats: | 798058 | 81893385 bp | | 6.13 % |
| Low complexity: | 82356 | 7335820 bp | | 0.55 % |

*most repeats fragmented by insertions or deletions have been counted as one element
