# Supplementary figures and images for: The Piranha Genome Provides Molecular Insight Associated to Its Unique Feeding Behavior
Source: Genome Biol Evol. 2019 Jul 8;11(8):2099–106. doi: 10.1093/gbe/evz139 (PMC6681833; doi:10.1093/gbe/evz139)

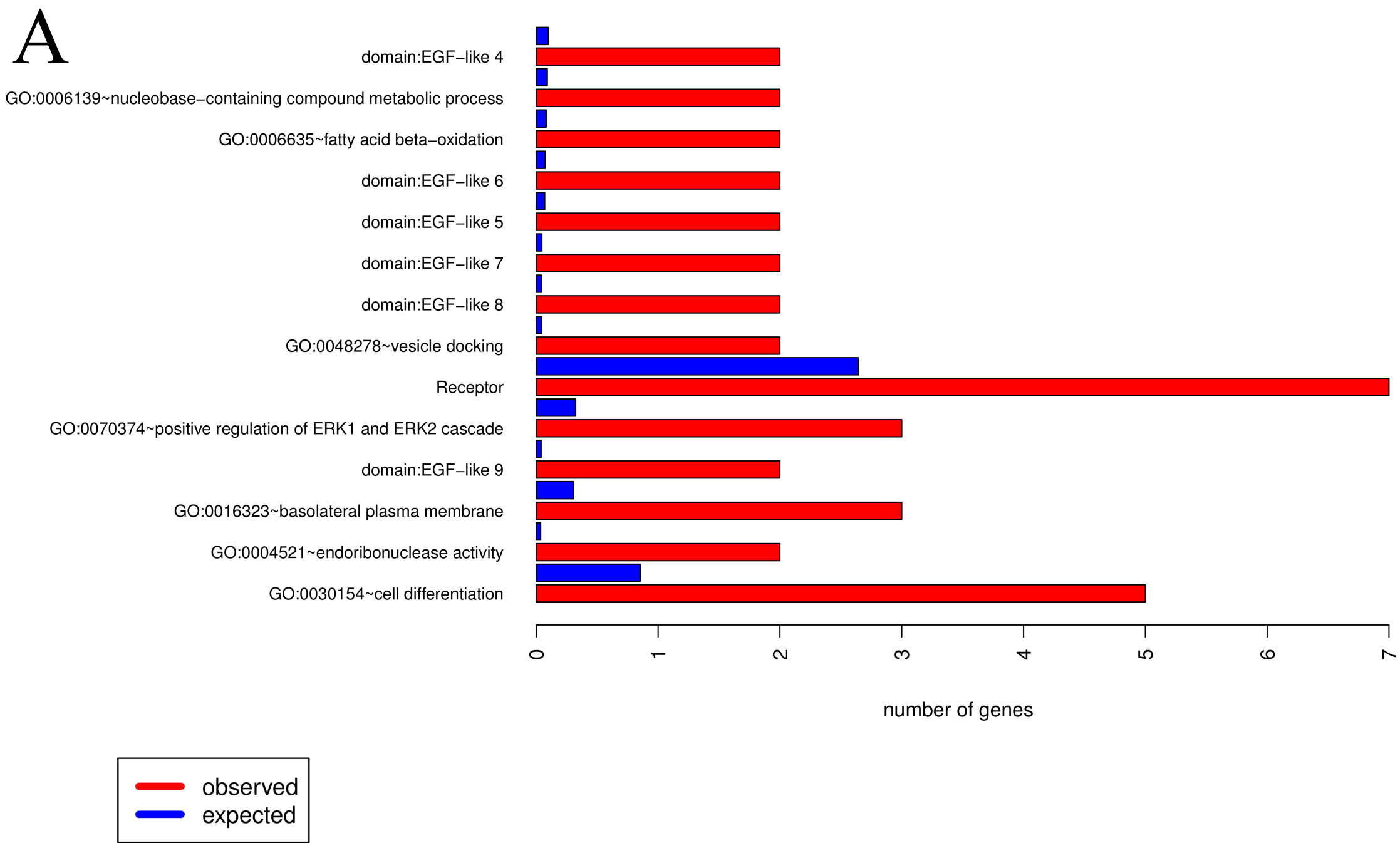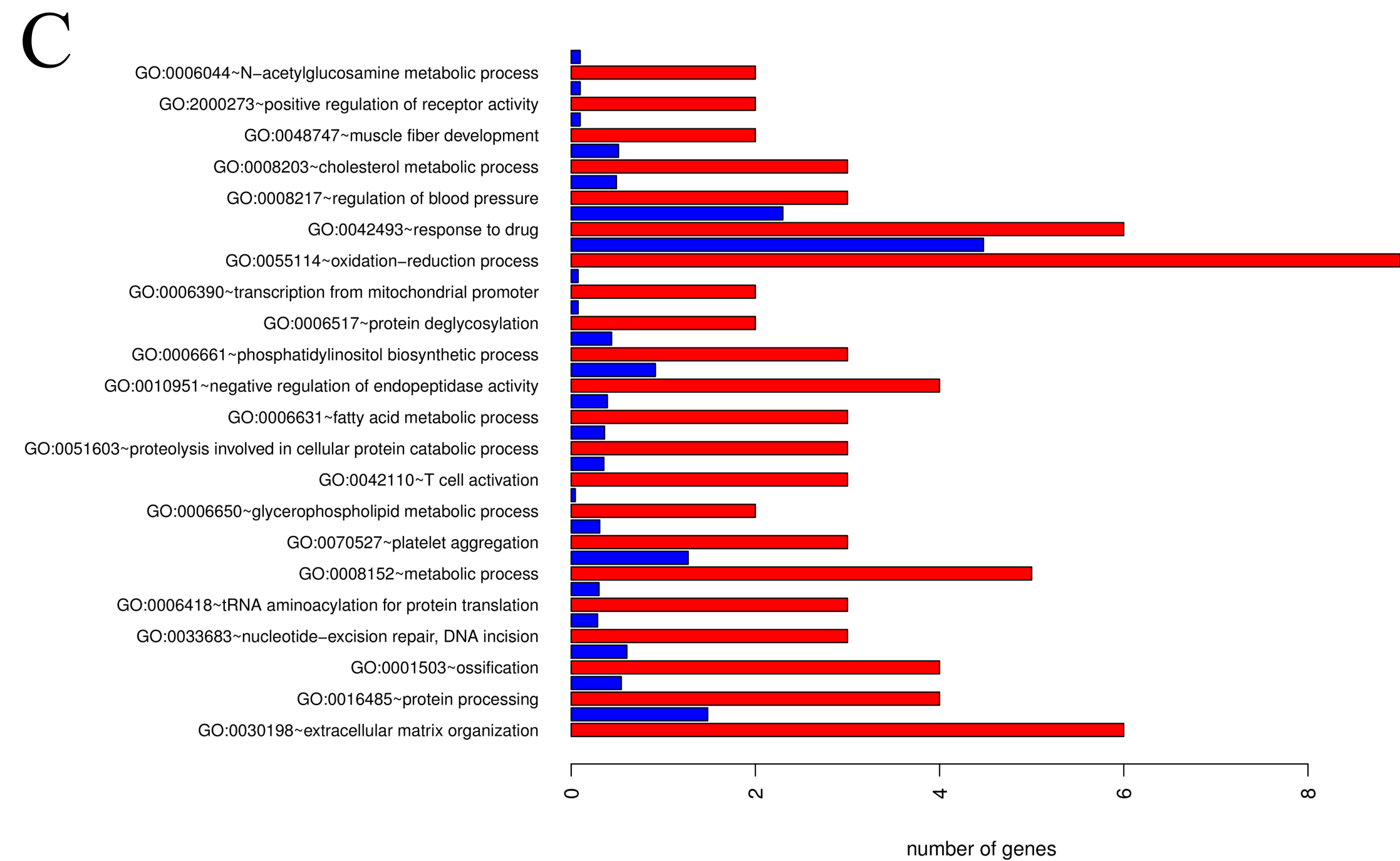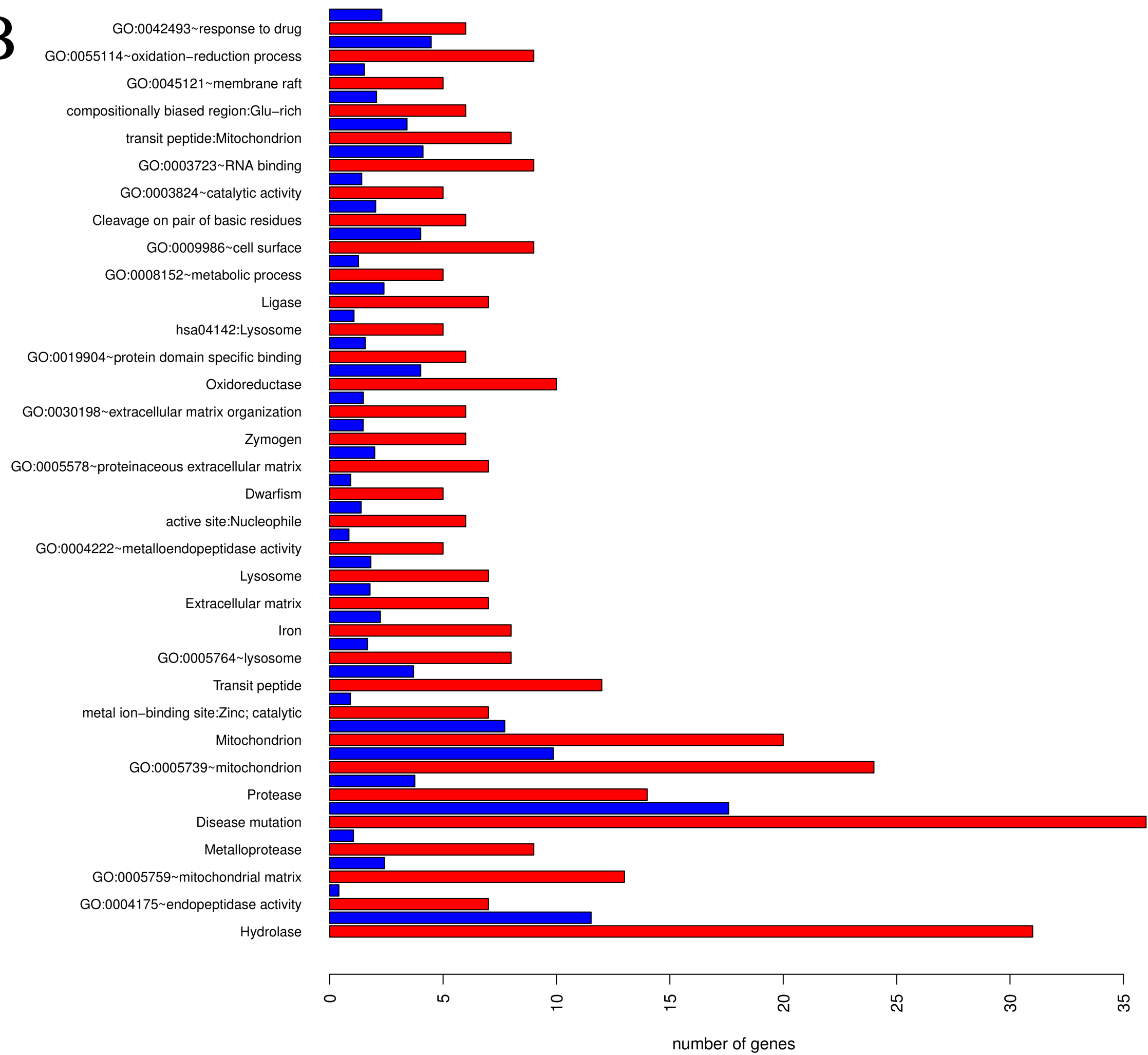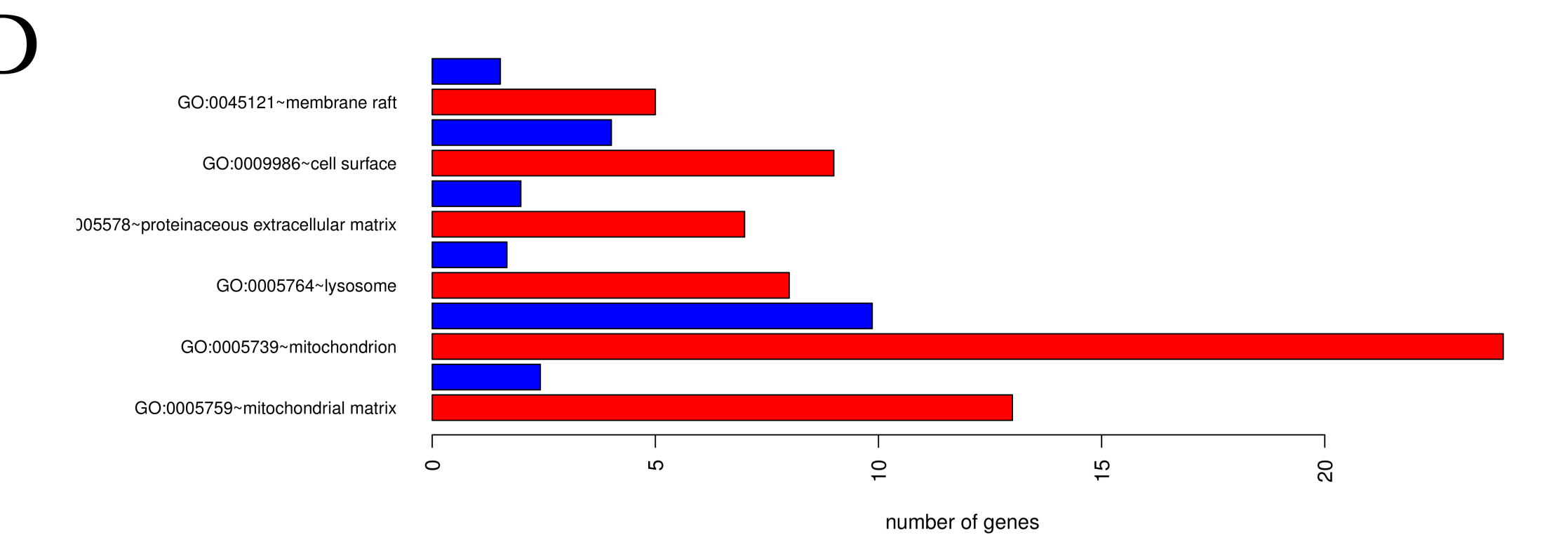

Supplement: Supplementary_Data_evz139 [file supplementary_data_evz139.zip › Supplementary Figure 1.pdf]

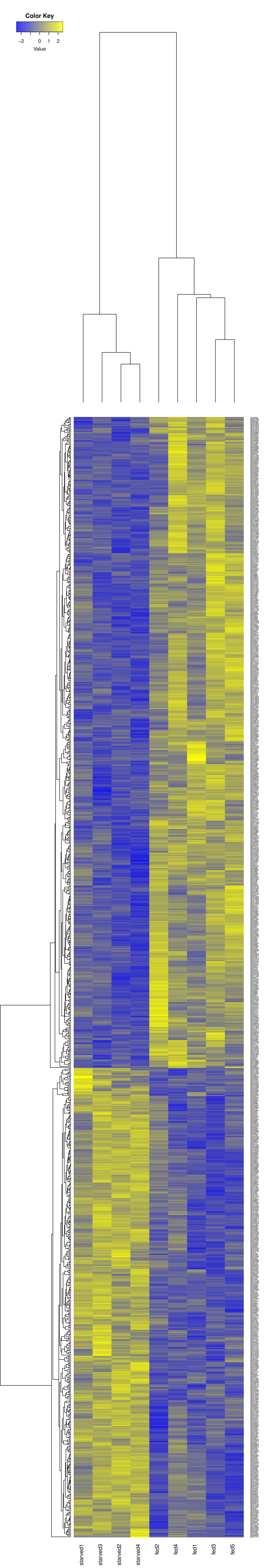

Supplement: Supplementary_Data_evz139 [file supplementary_data_evz139.zip › Supplementary Figure 3.pdf]

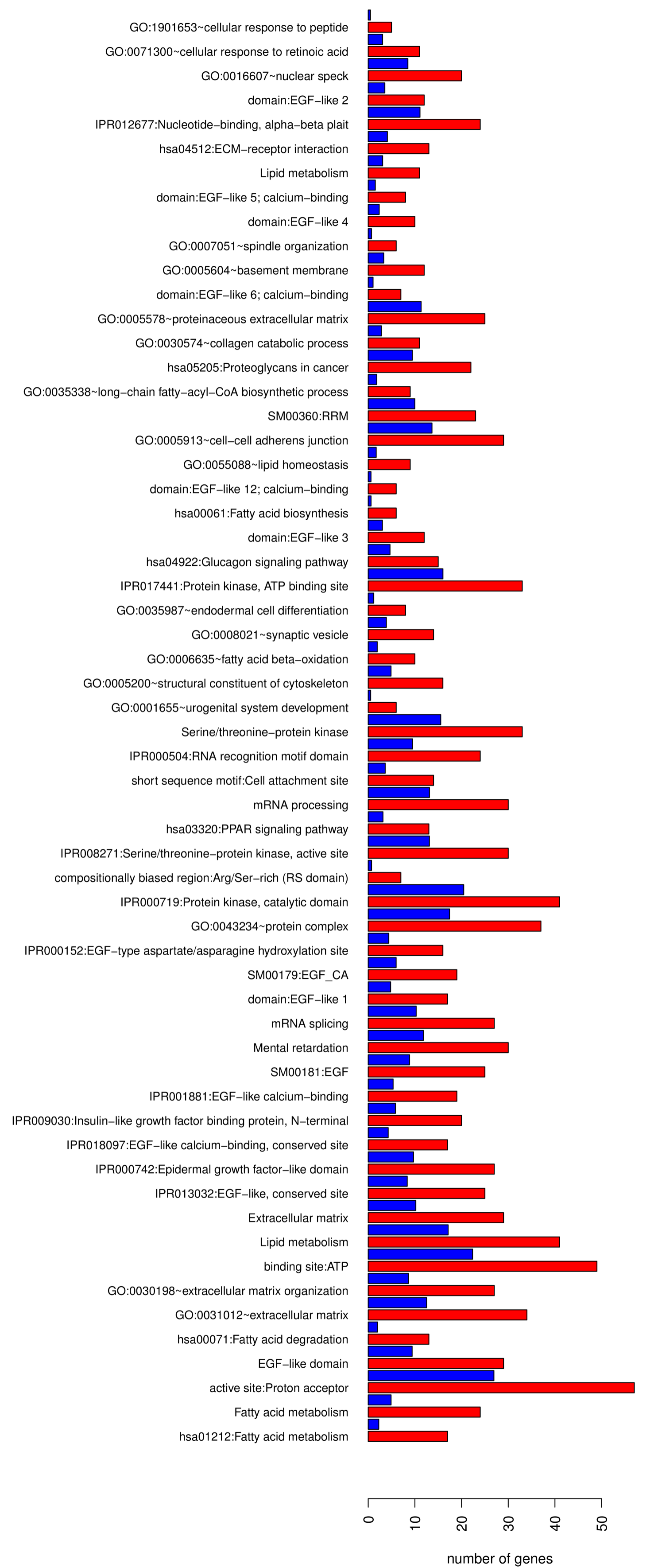

Supplement: Supplementary_Data_evz139 [file supplementary_data_evz139.zip › Supplementary Figure 4.pdf]

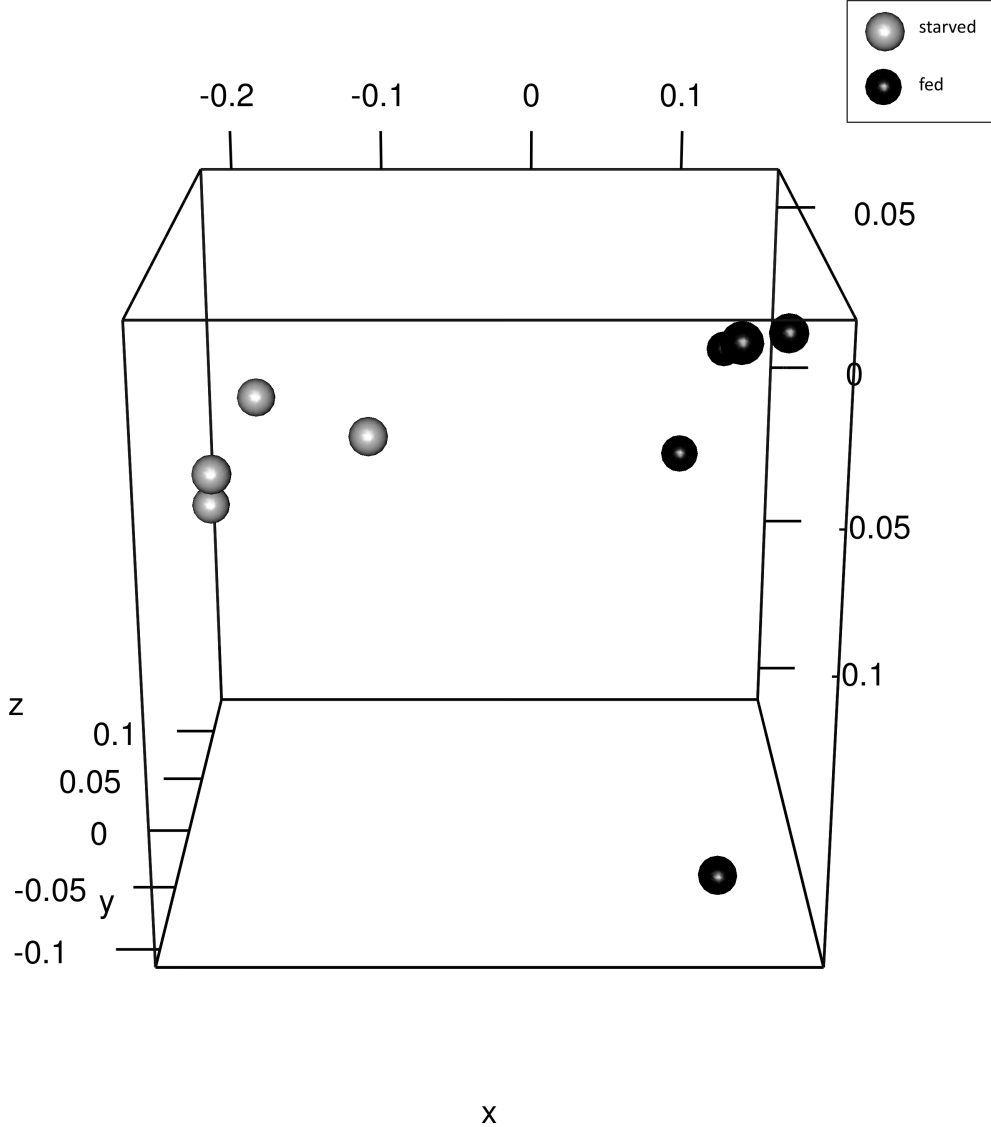

Supplement: Supplementary_Data_evz139 [file supplementary_data_evz139.zip › Supplmentary Figure 2 .pdf]
